# Successful management of refractory respiratory failure caused by avian influenza H7N9 and secondary organizing pneumonia: a case report and literature review

**DOI:** 10.1186/s12879-019-4306-7

**Published:** 2019-07-29

**Authors:** Hangyong He, Hao Wang, Xuyan Li, Xiao Tang, Bing Sun, Zhaohui Tong

**Affiliations:** Department of Respiratory and Critical Care Medicine, Beijing Institute of Respiratory Medicine, Beijing Key Laboratory of Respiratory and Pulmonary Circulation, Beijing Chao-Yang Hospital, Capital Medical University, Beijing, No. 8 Gongren Tiyuchang Nanlu, Chaoyang District, Beijing, (100020) China

**Keywords:** Organizing pneumonia, Avian influenza H7N9, Respiratory failure

## Abstract

**Backgroud:**

Organizing pneumonia (OP) is a rare complication of influenza infection that has substantial morbidity. We report the first case of OP associated with avian influenza H7N9 infection that had significant improvement with corticosteroid treatment.

**Case presentation:**

A 35-year-old male admitted to intensive care unit because of respiratory failure. He was diagnosed as severe pneumonia caused by avian influenza H7N9 viral infection. After initial clinical improvement supported by extracorporeal membrane oxygenation (ECMO), the patient’s condition worsened with persistent fever, refractory hypoxemia. Chest x-rays and computed tomographies showed areas of consolidation and ground glass opacification. Although OP was suspected and 1 mg/kg methylprednisolone was used, the patient’s condition didn’t improved considerably. An open lung biopsy was performed, and histopathological examination of the specimen was compatible with OP. The patient was treated with methylprednisolone 1.5 mg/kg for 5 days. ECMO was weaned on day 15, and he was discharged on day 71 with good lung recovery.

**Conclusions:**

To the best of our knowledge, this was the first case of successful management of refractory severe respiratory failure caused by avian influenza H7N9 infection complicated with OP. Refractory hypoxia with clinical manifestation and radiological findings compatible with OP, a differential diagnosis should be considered among patients at the second or third week of influenza H7N9 infection, especially in patients with clinical condition deteriorated after the primary influenza pneumonia was controlled. And a steroid dose of methylprednisolone 1.5 mg/kg may be suggested for treatment of OP associated with avian influenza H7N9 infection.

## Background

Human infected with avian influenza A H7N9 virus were first confirmed on March 30th, 2013 in China [1, 2], with high incidence of severe respiratory failure, high intensive care unit (ICU) admission and mortality. The development of organizing pneumonia (OP) has been reported in patients with influenza A, H1N1 and influenza B, and a high incidence of more than 10% of OP in influenza A H1N1 was reported in one case series [3–5]. In this report, we describe the first case of OP associated with avian influenza H7N9 infection.

## Case presentation

**Fig. 1:**
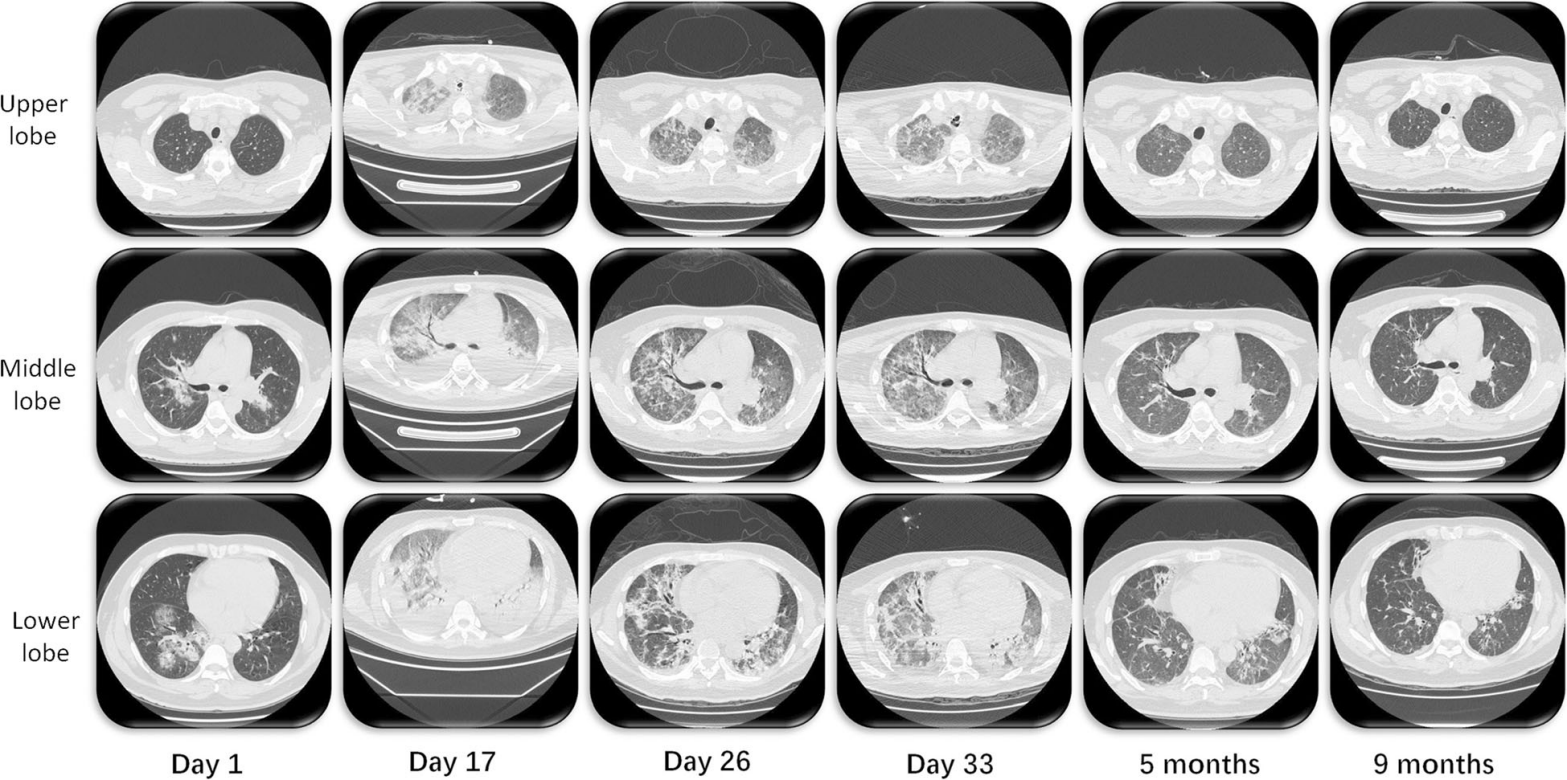
Chest computed tomography (CT) on day 1, day 17, day 26 and day 33 and followed up after 5 months and 9 months. Three representative slices of the upper, middle and lower lobe were chosen

A 35 year-old male, non-smoker, with a history of poultry contact 10 days before, was admitted to emergency room with fever and cough for 4 days (considered as day 1 for the case timeline). Physical examination showed bilateral moist crackles. Laboratory tests showed white blood cell count (WBC) was 5.75 × 10^9^/L (and it became to 1.35 × 10^9^/L two days later), C reactive protein (CRP) was 13.3 mg/L, and procalcitonin (PCT) was<0.1 ng/ml. Chest x-ray and chest computed tomography (CT) showed bilateral ground-glass opacities (GGO) and consolidation (Fig. 1). Moxifloxacin 400 mg daily was administered for two days. And his condition deteriorated with dyspnea and severe respiratory failure, and the blood gas analysis showed PaO_2_ was 58 mmHg under oxygen mask with a FiO2 of 0.8. He was transferred to our intensive care unit (ICU) supported with noninvasive ventilation (NIV) and intubated 3 h later. Mechanical ventilation with peak inspiratory pressure (PIP) of 32 cm H_2_O, positive end expiratory pressure (PEEP) of 20 cm H_2_O and FiO_2_ of 1.0 could not maintain the oxygenation. As his PaO_2_/FiO_2_ ratio less than 50 mmHg lasted for 3 h, the venovenous-extracorporeal membrane oxygenation (VV-ECMO) was established. The microscopic examination, culture and galactomannan detection from serum and bronchial-alveolar lavage fluid (BALF) for virus PCR, fungal, and the culture for bacteria and the microscopic examination for bacteria and tuberculosis were done at ICU admission. The nucleic acid polymerase chain reaction (PCR) for influenza H7N9 virus of sputum specimen turned out to be positive and oseltamivir phosphate was initiated (150 mg twice daily for 2 weeks after the PCR of the virus were negative for two consecutive tests.). As the PCT rose to 4.84 ng/ml, and the galactomannan detection was positive (2.52 from serum sample and 1.21 from BALF sample), vancomycin, imipenem cilastatin and caspofungin were applied. Hydrocortisone 300 mg daily for 3 days was also used for septic shock.

ECMO was weaning off on day 15 (the time of ECMO supporting was 13 days) when his blood flow of ECMO was decreased to less than 2 L/min with a significant improvement on his chest X-ray. The patient turned to high fever in the following days. Repeated PCR for H7N9 virus were tested and show continuous negative in the lower respiratory samples after a week of ICU admission. Advanced antibiotics and antifungal agents were administered, no positive pathogenic result was emerged, and PCT level remained downtrend. The chest CT on day 17 shows bilateral GGO with aggravating consolidation on new areas (Fig. 1), compatible with organizing pneumonia (OP). Considering no underlying cause of OP existed other than virus infection, therefore, OP associated with H7N9 influenza virus infection was suspected. Methylprednisolone 80 mg (1 mg/kg) daily was applied on day 17 for 5 days with tapering. With clinical improvement, the patient was extubated on day 21, and supported with NIV with a FiO_2_ of 0.6. The chest CT on day 26 showed obvious remission of consolidation with patchy GGO and fibrotic changes.

**Fig. 2:**
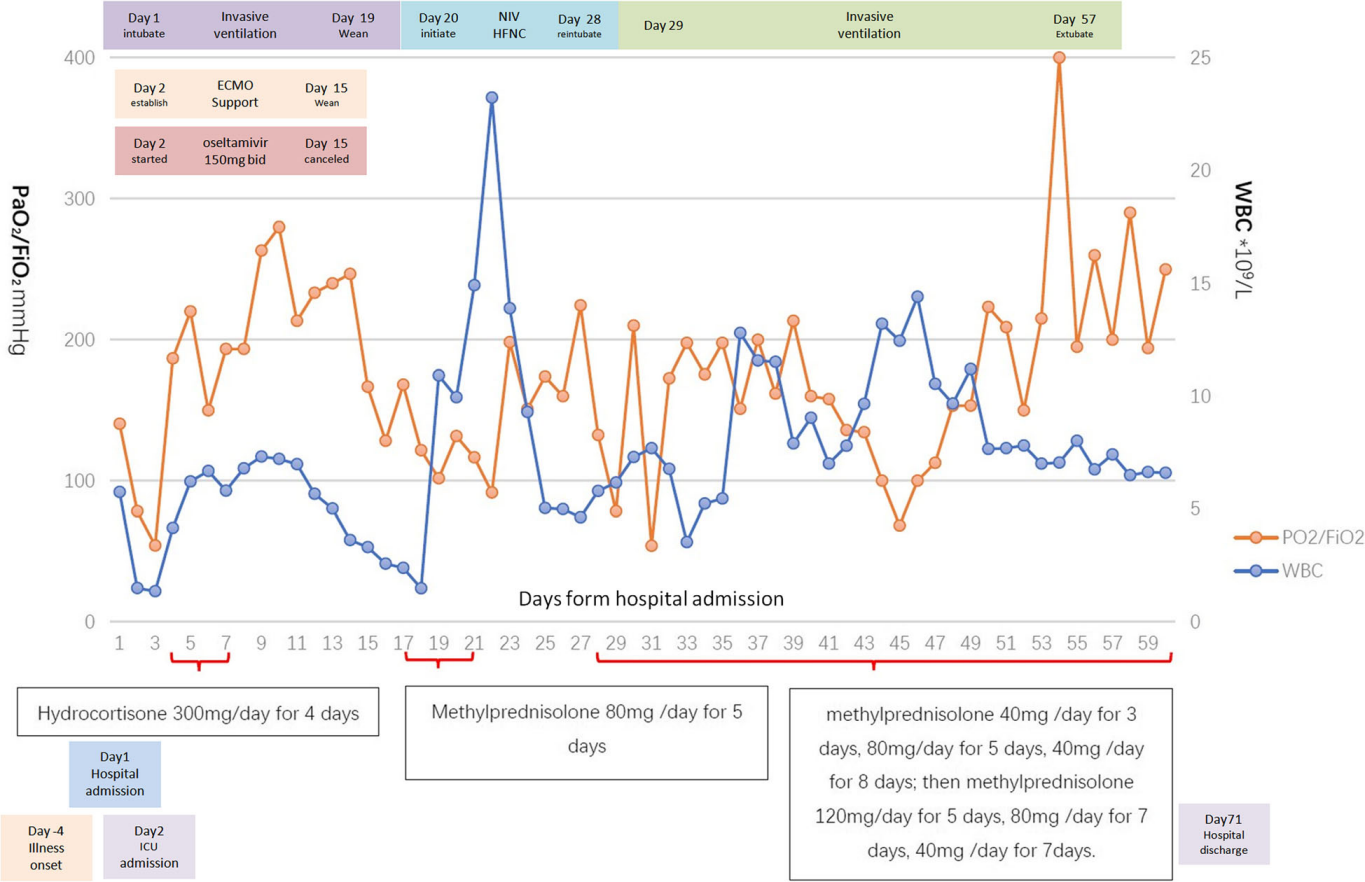
Time line of the steroids use, white cell count and ratio of PaO_2_/FiO_2_

However, the clinical condition of the patient deteriorated again on day 31 with high fever to 40 °C, refractory hypoxemia (PaO_2_:FiO_2_ = 65) and a mild leukopenia (WBC was 11.92 × 10^9^/L). the patient was reintubated and supported with invasive mechanical ventilation. Methylprednisolone 80 mg daily was applied at the beginning as a suspicion of the relapse of OP. And chest CT on day 33 revealed progression of consolidation especially in the lower lobe. As the patient’s respiratory failure and condition did not improve after 5 days of daily use of methylprednisolone 80 mg, histological examination was done via open lung biopsy (OLB) on day 35, and OP was confirmed with the presence of intraluminal plugs of granulation tissue within alveolar ducts and surrounding alveoli associated with chronic inflammation of the surrounding lung parenchyma. The therapy of steroid was changed to methylprednisolone 120 mg (1.5 mg/kg) for 5 days, 80 mg for 7 days, 40 mg for 7 days. The oxygenation improved, and the patient was extubated on day 57 and discharged on day 71. A time line of the steroids use, white cell count and ratio of PaO_2_/FiO_2_ is illustrated in Fig. 2.

The following-up for 10 months from onset of primary virus infection showed gradually improvement, with mild interlobular septal thickening, traction bronchiectasis and consolidation in chest CT on the ninth month (Fig. 1).

### Literature review

As shown in Tables 1, 13 previously published cases and the current case of OP associated with influenza virus infection were reviewed [3–10]. With available information, the age range was 24-year-old to 97-year-old with 64% (9/14) female. Influenza A constituted the majority (93%,13/14), with 8 cases were identified to H1N1, and the current case, H7N9. The other one case was OP complicated with co-infection of influenza B and Streptococcus. Respiratory failure associated with OP was reported in 64% (7/11) patient. Fever (27%, 3/11), dyspnea (36%, 4/11), cough (27%, 3/11) were the most common symptoms reported. Especially, clinical condition deteriorated after controlling of primary disease can be found in 64% (7/11) patients. GGO and consolidation were the main findings on high resolution computed tomography (HRCT), shown in 50%(6/12) and 67%(8/12) of cases, with release of primary opacity associated with influenza infection in some cases. Autopsy were applied at about two weeks and most transbronchial lung biopsy (TBLB) or open lung biopsy (OLB) were applied over three weeks. Steroid was the main treatment, varying from prednisolone 30 mg/day to methylprednisolone 500 mg/day pulse therapy, with or without tapering. Most patients react well to the treatment with clinical and radiological improvement, excepting the relapsing of OP in our case.

**Table 1:** Cas series reported organizing pneumonia associated with influenza viral infection

Citation	Age	Gender	Virus type	Manifestation	HRCT	Time to biopsy	Biopsy/autopsy	Pathology	Treatment	Outcome
Staud, 2001 [4]	59	F	A	Respiratory failure	Consolidation	NR	OLB	OP	Intravenous steroids and azathioprine	Survival
Fujita, 2007 [6]	38	F	A	NR	Consolidation	NR	NR	OP	Steroid pulse therapy	Survival
Fujita, 2014 [7]	97	F	A	Respiratory failure	Not examined	12 days	Autopsy	OP, DAD, hemorrhage and bronchiolitis	NR	Dead
	24	F	H1N1	Respiratory failure	Consolidation and GGO	16 days	Autopsy	OP and DAD	NR	Dead
	37	M	H1N1	Respiratory failure	GGO	15 days	Autopsy	OP, hemorrhage and edema	NR	Dead
Marchiori, 2011 [8]	52	M	H1N1	NR	linear opacity	NR	NR	OP	NR	NR
Torrego, 2010 [9]	55	F	H1N1	Dyspnea and cough	Consolidation	Over 30 days	TBLB	OP with viral cytopathic changes	Prednisone, 0.75 mg/kg/d	C/R improvement
Cornejo, 2010 [3]	52	F	H1N1	Respiratory failure	Consolidation	Over 8 days	OLB	OP, bronchiolar necrosis and squamous metaplasia	Methylprednisolone, 500 mg/d for 3d	improvement
	36	M	H1N1	Fever, increased inflammatory parameters, and respiratory failure	Consolidation and loss of global lung volume	Over 3 weeks	OLB	OP, hemorrhage and edema	The same as above	C/R improvement
Gómez-Gómez, 2011 [10]	44	F	H1N1	Cough, dyspnea and fever	Intralobular interstitial thickening and GGO	Over 3 weeks	TBLB	OP	Steroid, 1 mg/kg/day for 1 m 0.5 mg/kg/d for 6w	C/R improvement
	60	M	H1N1	Cough and dyspnea	Consolidation and GGO	Over 3 weeks	TBLB	OP	The same as above	C/R improvement
Kwok, 2016 [5]	45	F	B	Dyspnea	GGO and lung cyst	Over 28 days	TBLB	OP	Prednisolone, 30 mg/d with 2 m tapering	C/R improvement
	NR	F	A	NR	NR	NR	NR	NR	Steroid	Improvement with lung fibrosis and residual dyspnea
This case	35	M	H7N9	Fever, respiratory failure	Consolidation and GGO	39 days	OLB	OP, hemorrhage and edema	Methylprednisolone, 1.5 mg/kg/d for 5d,	C/R improvement

## Discussion and conclusions

Since the cases infected with avian influenza H7N9 virus were first confirmed in 2013 in China [1, 2], five seasonal epidemics were observed, with an earlier start and a steep increase in infected number in the latest epidemic [11]. In a clinical study including 111 cases confirmed H7N9 virus infection, 76.6% were admitted to an ICU and 27% died. The median time from the exposure to disease onset was five days, then, 6.8 days to acute respiratory distress syndrome (ARDS). In dead cases, the median time from disease onset to death was 14 days [12]. Half of the patients required mechanical ventilation and 21% need ECMO for severe respiratory failure [1, 2]. However, refractory severe respiratory failure caused by OP secondary to avian influenza H7N9 virus infection was first reported in this case.

OP was originally described by Davison et al. in 1983 [13] (1), and in 1985, Epler et al. described the same entity as the term “bronchiolitis obliterans organizing pneumonia” (BOOP) [14]. Now, for avoiding confusion with bronchiolitis obliterans (BO), the term organizing pneumonia (OP) is preferred [15]. In fact, OP is an non-specific inflammatory response from human body towards acute lung injuries. It can be with idiopathic nature, namely cryptogenic organizing pneumonia (COP), or be associated with different clinical settings, such as infections (bacteria, virus, fungus or parasite), drugs (antibiotics, antiepileptics, immunomodulators), connective tissue disease, vasculitis or lung/marrow transplantation. Considering symptomatology, physical signs, laboratory and pulmonary function tests, radiologic or histomorphological findings, there is no obvious difference between COP and secondary OP, excepting the latter may have higher mortality [16].

OP has been linked to multiple influenza viral infections including influenza A and B, but this is the first report of avian influenza H7N9-associated OP with relapse occurrence and severe respiratory failure. The main clinical features of our avian influenza H7N9-associated OP is similar to the cases of influenza A cases. In these cases reported, OP onset mostly at the second to third week in the course of influenza, and occurred after the releasing of primary virus infection; And the OP is complicated with respiratory failure, and no evidence of other pathogen was found; And the main findings on HRCT for this kind of OP were GGO and consolidation. Biopsy were done via TBLB or OLB at the third week. In cases with influenza associated OP, other than common findings of OP, diffuse alveolar damage (DAD), alveolar hemorrhage and edema and bronchiolitis can also be found, showing the lung injury of primary virus infection. However, in our case, the patient present with high fever as a main manifestation of OP, and had a relapse of respiratory failure associated with OP. A differential diagnosis of OP should be considered at the second to third week after the primary infection among patients with influenza H7N9 infection. Furthermore, there was no clear evidence of bacterial and fungus infection during the beginning of hospitalization. However, the levels of PCT and galactomannan showed significant increase after the patient was established with ECMO. The OP may also be caused by the nosocomial infection which was frequently complicated with severe influenza pneumonia.

The majority of patients with OP show rapid responding to steroids. The introduced initial dose is prednisone 0.5–1.0 mg/kg, with tapering over 6–12 month. However, up to one-third patients may relapse in tapering period. For the current patient with avian influenza H7N9-associated OP, methylprednisolone 80 mg (1 mg/kg) daily was applied for 5 days with tapering in the beginning of suspicion of OP. The patient showed rapid clinical and radiological improvement and was extubated on the fifth day of steroids applying. However, the patient deteriorated with high fever and refractory hypoxemia 10 days later. With the confirmation of histological examination, steroid dose was increased to methylprednisolone 120 mg (1.5 mg/kg) daily for 5 days. And OP was finally controlled without relapsing in follow-ups. Insufficient initial dose of steroid may contributed to the relapsing of OP before final diagnosis. Therefore, Post avian influenza H7N9 infection OP may responsive to a brief course (weeks) of moderate-to-high dose prednisone therapy.

To the best of our knowledge, this was the first case of OP associated with avian influenza H7N9 infection. With clinical manifestation and radiological findings compatible with OP, a differential diagnosis should be considered among patients with influenza H7N9 infection at the second or third week after the initial viral infection, especially in patients with clinical condition deteriorated after controlling of primary influenza pneumonia. And a steroid dose of methylprednisolone 1.5 mg/kg maybe suggested for treatment of OP associated with avian influenza H7N9 infection. Our case provide clinical insight into refractory respiratory failure with lung involvement due to avian influenza H7N9. OP should be considered on the differential diagnosis of patients with fever and respiratory failure after severe influenza A H7N9 infection, where steroids might be useful.

## Data Availability

Not applicable.

## Abbreviations

ARDS: Acute respiratory distress syndrome
GGO: Ground-glass opacity
ICU: Intensive care unit
OLB: Open lung biopsy
OP: Organizing pneumonia
